# Critical Analysis of Reporting Quality of Network Meta-Analyses in Periodontology and Implantology

**DOI:** 10.1055/s-0044-1801304

**Published:** 2025-03-12

**Authors:** Heba Mahmoud Ashi, Zohaib Khurshid

**Affiliations:** 1 Department of Dental Public Health, Faculty of Dentistry, King Abdulaziz University, Jeddah, Saudi Arabia; 2 Department of Prosthodontics and Dental Implantology, College of Dentistry, King Faisal University, Al-Ahsa, Saudi Arabia; 3 Center of Excellence for Regenerative Dentistry, Department of Anatomy, Faculty of Dentistry, Chulalongkorn University, Bangkok, Thailand

**Keywords:** periodontology, implantology, systematic reviews, network meta-analysis, quality analysis and evidence-based dentistry

## Abstract

The increasing recognition of network meta-analyses (NMAs) in dentistry, particularly in periodontology and implantology, lacks assessed reporting quality. To address this, our study will undertake a systematic review of previously reported NMAs. Researchers conducted an electronic search in Web of Science and Scopus to identify NMAs across all dentistry journals. Two independent investigators selected studies, extracted data, and assessed reporting quality using the Preferred Reporting Items for Systematic Reviews and Meta-Analyses for NMA (PRISMA-NMA) checklist with 32 items. Each “yes” response earned 1 point, and “no” responses received 0 points, yielding an overall reporting quality score. In total, 39 NMAs were included in this study. The NMAs were published between 2010 and October 2024, with most of them published in 2022 (25%). Most of the NMAs employed the PRISMA-NMA guidelines (47%) and have been published in the *Journal of Clinical Periodontology* (53%). The overall reporting quality of the included NMAs ranged between 87.5 and 100% (i.e., high quality of reporting [≥ 75th %]), with 5 NMAs reporting all 27 items of the PRISMA-NMA statement. The limitations, presentation of network structure ( *results* ), funding, and objectives ( *methods* ) were reported in 97, 94, 81, and 78% of the NMAs, respectively. The least reported items were the protocol registration and the summary of network geometry, which were reported in 53% of the NMAs. All the remaining items were reported in all 39 NMAs. The reporting quality of the NMAs published related to periodontology and implantology was high. However, some deficiencies were revealed associated with the reporting quality of the PRISMA-NMA items, including protocol registration, formulation of the research question based on the PICO (Population, Intervention, Comparison, Outcome) format, and summarization of the network geometry.

## Introduction

Systematic reviews and meta-analyses (SR-MA) of randomized controlled trials (RCTs) are regarded as the highest level of research evidence for shaping policies and informing practical applications. 1 Nevertheless, a SR-MA may have limitations in evaluating treatment effectiveness solely through direct comparisons between treatments. In some cases, the absence of high-quality evidence could be attributed to the frequent lack of direct evidence. 2 This issue can potentially be solved by the application of a recently introduced approach to perform statistical analysis known as network meta-analysis (NMA), 3 which has gained recent popularity in the field of dentistry. 4 5 6 7 8

NMAs enable a quantitative synthesis of the studies that investigate various treatment sets. 9 This involves merging both direct as well as indirect evidence of treatments, established through a common set of comparators. 9 Compared with pairwise meta-analyses, NMAs integrate all accessible evidence within a unified statistical model, allowing for the visualization of a greater volume of evidence. This enables the estimation of relative efficacy and the grading of interventions across all treatments, despite the deficiency of direct head-to-head comparisons. 10

Empirical investigations, nevertheless, assessing the features of published NMAs of treatments have raised the requirement to improve the quality of NMA methods' application. 11 12 Issues regarding the inadequate utility of NMA methods as well as nontransparent and inappropriate reporting of methodology and outcomes have been detected as primary concerns. 13 Recently, Nagendrababu et al 14 concluded that no NMA included in endodontology sufficiently covered all the items in the Preferred Reporting Items for Systematic Reviews and Meta-Analyses for NMAs (PRISMA-NMA) checklist. Additionally, Petropoulou et al 15 highlighted substantial limitations in both the reporting and execution of numerical outcomes and statistical analysis in numerous NMAs found in medical literature.

Inadequate methodological and reporting quality in SRs can lead to skewed or erroneous results. It also poses challenges in terms of readability, dependability, transparency, and reproducibility, ultimately hindering the effective utilization of SRs in appropriate health care settings. 16 17 The PRISMA statement was published in 2009 (PRISMA-2009) to guide authors when reporting conventional pairwise SRs and meta-analyses. 18 This statement comprised a checklist having a flowchart and 27 items. PRISMA-2020 has replaced and updated this statement. 19 The extension of PRISMA-2009 was made in 2015 for including the reporting of SRs comparing multiple interventions utilizing indirect and direct evidence in NMAs of health care treatments. 20 The PRISMA for NMA (PRISMA-NMA) statement comprises a flowchart and checklist having 32 items (5 NMA items + 27 general items). 20

In the field of dentistry, there has been a consistent increase in the number of published NMAs over the past few years with a majority of them focusing on periodontics and implantology. However, to the authors' knowledge, no study has been conducted so far to evaluate whether the NMAs performed in periodontology and implantology are adequately reported. Therefore, this current study is unique in its focus, aiming to evaluate the reporting quality of NMAs published in the fields of periodontics and implant dentistry. This assessment involves conducting a SR of published NMAs to determine whether these studies appropriately adhered to the key reporting components outlined in the SR protocol based on the PRISMA-NMA statement. 20

## Methods

### Study Search Strategy and Selection Protocol

For this review 92 journals categorized under “Dentistry, Oral Surgery, and Medicine” from the 2021 Journal Citation Reports Science Edition—a section of Clarivate Analytics' Web of Science ( https://www.jcr.clarivate.com ))—were selected. Subsequently, an electronic search was conducted in Elsevier's Scopus ( https://www.scopus.com ) from their inception until February 28, 2023, which was updated on October 28, 2024. The search terms “network meta-analysis” or “indirect meta-analysis” were queried in the “article title” section. The search also included terms such as “mixed treatment meta-analysis,” “mixed intervention meta-analysis,” “multiple treatment comparison meta-analysis,” “multiple intervention comparison meta-analysis,” “bayes meta-analysis,” and “indirect comparison.” For screening purposes, “dentistry” was selected as the subject area. A similar search was conducted on PubMed, ISI Web of Science, and Google Scholar. We included studies that were NMA of RCTs focusing on patients treated in the fields of periodontics and dental implants. There was no restriction on the interventions, comparators, and outcomes. All SRs with NMAs published in English related to periodontology and implantology were considered to be included in the present study. H.M.A. and Z.K., both independent authors, evaluated the titles and abstracts of each retrieved article to establish its eligibility. Afterwards, researchers sought for the complete texts of papers that may have been pertinent for additional analysis. Each disagreement between the two researchers was discussed and resolved verbally until they came to an agreement. The sample search strings for all databases used for this review are provided in Supplementary Material (available in the online version only).

### Data Tabulation

Each study's data was retrieved using the following variables: (1) the name of the first author; (2) the year of publication; (3) the nation of both the first and corresponding authors; (4) the name of the journal; (5) the kind of reporting criteria; (6) the number of citations according to Scopus; (7) the funding source; and (8) the numbers of primary or main articles included in the NMA.

### Pilot Study

For increasing the accuracy of the extracted data as well as evaluating NMAs' quality, a pilot study was performed by two primary assessors (H.M.A. and Z.K.) on five of the retrieved NMAs. The primary assessor (H.M.A.) randomly selected five studies for this purpose. At this stage, disagreements between the two assessors were resolved by discussion.

### Evaluation of Reporting Quality

The reporting quality was evaluated utilizing the PRISMA-NMA guideline, which consists of 27 items ( Supplementary Table S1 , available in the online version only). 20 A score of “yes” denoting full or sufficient compliance and “no” denoting noncompliance were assigned to each item on the PRISMA-NMA checklist to assess the level of adherence. According to the scoring procedure, one point was given for each “yes” and zero points for each “no” response; the sum of these points was used to determine the overall reporting quality score. 16 The resulting overall PRISMA-NMA scores were subsequently categorized into three distinct groups: (1) high (i.e., ≥ 75th percentile), (2) medium (i.e., the interquartile range), and (3) low (i.e., ≤ 25th percentile). 21 For each item, percentage scores were categorized as: (1) poor (i.e., 90%). 22 The total reporting quality score for each NMA was further converted into a percentage using the following formula:

Overall reporting quality score = total number of “Yes”/32 × 100

## Results

### Literature Search Outcomes

**Fig. 1:**
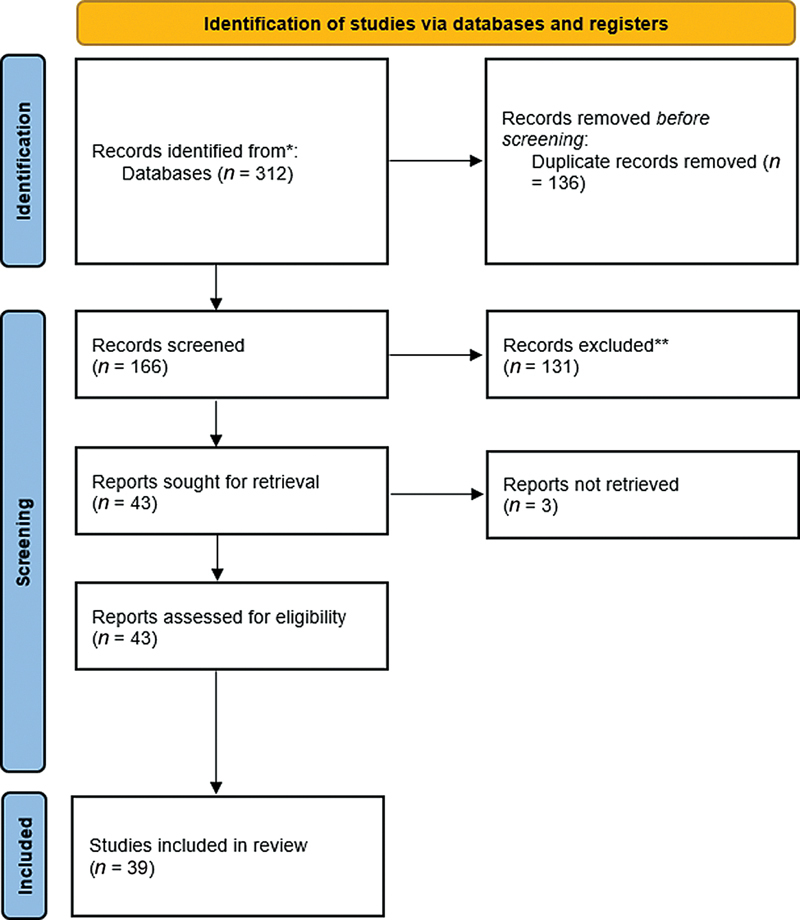
Flowchart of the literature search strategy according to the Preferred Reporting Items for Systematic Reviews and Meta-Analyses (PRISMA) guidelines.

A flow diagram representing the methodology for searching the literature is shown in Fig. 1 . There were 312 articles found in the initial search. After 136 duplicate articles were removed, 131 publications that were not related to periodontology and/or implantology were excluded. Of these 43 articles, the full-text of 6 articles 22 23 24 25 26 27 was not available online, hence, the corresponding authors of the respective studies were contacted via email. The corresponding authors of three studies responded with the full-texts, 22 23 24 while the authors of the present study were not able to get any response from the corresponding authors of the remaining three studies. 25 26 27 These 39 papers were included in the present study after their full-texts were evaluated and found to meet the eligibility criteria ( Supplementary File , available in the online version only).

### Primary Characteristics of the Included Studies

The main features of the 39 NMAs that were included are shown in Table 1 . The studies outlined in the document each focus on distinct objectives within dental implantology and periodontics, primarily evaluating treatment effectiveness, comparing approaches, or synthesizing evidence using NMAs. For instance, Aldhohrah et al sought to examine the impact of different overdenture attachments on peri-implant tissue health in a study involving 599 patients, while Camps-Font et al investigated the influence of implant abutment connections on implant-supported prostheses, enrolling 1,042 patients. Other studies, such as those by Bi et al with 1,166 patients and Rabelo et al with 388 patients, similarly focus on the effectiveness of implant-supported overdentures and antibiotic therapy for periodontitis, respectively. The sample sizes vary widely across studies, reflecting the range of research designs and specific aims within this field.

**Table 1:** General characteristics of the included studies

Study	Country of correspondence – 1 ^st^ author	Journal title	Reporting guidelines used in the study	Citation count (Scopus)	Funding source	Articles included in the NMA	Objective	No. of patients included
(Aldhohrah et al. 2022)	China – China	*Journal of Prosthetic Dentistry*	PRISMA-NMA	5	NR	16	To evaluate the effect of different overdenture attachments with delayed or immediately loaded 2-implant-retained mandibular overdentures on peri-implant tissue health.	599
(Camps-Font et al. 2021)	Spain – Spain	*Journal of Prosthetic Dentistry*	PRISMA-NMA	10	No funding	18	To assess whether the implant abutment connection influences the outcome of implant-supported prostheses	1042
(Hu et al. 2019)	China – China	*Journal of Dentistry*	PRSMA-IPD	14	National Natural Science Foundation of China	23	To compare the survival rate of abutments, marginal bone loss and peri-implant soft tissue discoloration among implant-supported single crowns with different abutment materials.	NR
(Bi et al. 2022)	China – China	*Journal of Prosthodontic Research*	PRISMA-NMA	0	Guangzhou Science & Technology; Department of Education of Guangdong Province; high-level university construction funding of Guangzhou Medical University	28	To evaluate the effect of overdenture (OD) attachment type and the number of implants supporting mandibular ODs on peri-implant health	1166
(Severi et al. 2022)	Italy – Italy	*Clinical Implant Dentistry and Related Research*	PRISMA	4	Universita degli Studi di Ferrara	24	to evaluate the effect of different lateral bone augmentation (LBA) procedures on the complete correction of a peri-implant bone dehiscence (BD) or fenestration (BF) from implant placement to implant surgical uncovering	NR
(Faggion Jr et al. 2013)	Germany – Germany	*Clinical Implant Dentistry and Related Research*	NR	61	Medical Faculty of the University of Heidelberg; UK government's Higher Education Funding Council for England	11	To demonstrate the application of network meta-analysis in implant dentistry using peri-implantitis treatment as an example.	NR
(Panda et al. 2022)	Italy – Italy	*Clinical and Experimental Dental Research*	PRISMA	1	Yes, but not mentioned	39	To rank different biomaterials used in adjunct to coronally advanced flap (CAF), based on their performance in rootcoverage for Miller's Class I and II gingival recessions.	858
(Li et al. 2022)	China – China	*International Journal of Oral Maxillofacial Implant*	PRISMA-NMA	2	National Natural Science Foundation of China; Construction Engineering Special Fund of “Taishan Scholars” of Shandong Province; Open Foundation of Shandong Provincial Key Laboratory of Oral Tissue Regeneration; Dean's Foundation of the 960th PLA Hospital	11	to compare the clinical effects of nonaugmentative adjunctive approaches in the surgical treatment of peri-implantitis.	NR
(Tu et al. 2010)	UK – UK	*Journal of Clinical Periodontology*	NR	63	Medical Faculty of the University of Heidelberg; UK government's Higher Education Funding Council for England	28	To investigate whether combination therapy yields better clinical outcomes in the treatment of infrabony defects compared with the use of EMD alone	NR
(Buti et al. 2013)	Italy – Italy	*Journal of Clinical Periodontology*	PRISMA	75	Yes, but not mentioned	31	To conduct a Bayesian network meta-analysis(NM) of randomized controlled trials (RCTs) to establish a ranking in efﬁcacy and the best technique for coronally advanced ﬂap (CAF)-based root coverage procedures	NR
(Tu et al. 2012)	UK – UK	*Journal of Clinical Periodontology*	NR	104	Medical Faculty of the University of Heidelberg; UK government's Higher Education Funding Council for England	53	To conduct a Bayesian network meta-analysis of randomized con-trolled trials on treatment eﬀects of GTR, EMD and their combination therapies	NR
(Faggion Jr et al. 2014)	Germany – Germany	*Journal of Clinical Periodontology*	PRISMA	62	National Science Council in Taiwan	11	To compare the clinical effect of various non-surgical peri-implantitis therapies	361
(Rabelo et al. 2015)	USA – Brazil	*Journal of Clinical Periodontology*	PRISMA	73	Sao Paulo Research Foundation; National Science Council in Taiwan	14	To assess the effect of systemic antibiotic therapy on the treatment of aggressive periodontitis	388
(Escribano et al. 2016)	Spain – Spain	*Journal of Clinical Periodontology*	PRISMA-NMA	40	Etiology and Therapy of Periodontal Diseases Research Group, University Complutense, Madrid, Spain.	51	To compare the efﬁcacy of different anti-plaque chemical agents, in 6-month, home-use, randomized clinical trials (RCTs), in terms of plaque index (PlI) changes	8810
(Iocca et al. 2017)	Italy – Italy	*Journal of Clinical Periodontology*	NR	63	Self-funded	6	To conduct a traditional meta-analysis and a Bayesian Network meta-analysis tosynthesize the information coming from randomized controlled trials on different socket grafting materials and combine the resulting indirect evidence in order to make inferences on treatments that have not been compared directly	NR
(John et al. 2017)	USA – USA	*Journal of Clinical Periodontology*	PRISMA	33	Ora-Pharma and Atrix Laboratories	74	The recent ADA-commissioned Clinical Practice Guideline on the nonsurgical treatment of chronic periodontitis has provided the most exhaustive library of clinical trials on scaling and root planing (SRP) with or without adjuncts. This network meta-analysis compared the adjuncts against each other.	NR
(Figuero et al. 2019)	Spain – Spain	*Journal of Clinical Periodontology*	PRISMA-NMA	33	Etiology and Therapy of Periodontal Diseases Research Group, University Complutense, Madrid, Spain.	53	to compare the efficacy of different oral hygiene products for chemical biofilm control, in 6-month home-use, randomized clinical trials (RCTs), in terms of changes in gingival index (GI)	8457
(Jepsen et al. 2020)	Germany – Germany	*Journal of Clinical Periodontology*	PRISMA-NMA	32	NR	19	To investigate the clinical performance of regenerative periodontal surgery in the treatment of furcation defects versus open flap debridement (OFD) and to compare different regenerative modalities	575
(Romandini et al. 2019)	Italy – Italy	*Journal of Clinical Periodontology*	PRISMA-NMA	49	NR	8	To answer to the following question: "In patients undergoing dental implant placement, which is the best antibiotic prophylaxis protocol to prevent early failures?"	1693
(Slot et al. 2020)	The Netherlands – The Netherlands	*Journal of Clinical Periodontology*	PRISMA-NMA	28	Regular academic appointments of Slot and Van der Weijden at the Academic Centre for Dentistry Amsterdam	16	To synthesize the available clinical evidence concerning efficacy of mechanical oral hygiene devices in periodontal maintenance patients	657
(Stavropoulos et al. 2021)	Switzerland – Switzerland	*Journal of Clinical Periodontology*	PRISMA	25	ASTI, a research consortium financed partly from the Danish Innovation Foundation.	30	To systematically assess the literature to answer the focused question “In periodontitis patients with intrabony defects, what are the medium- and long-term benefits of periodontal regenerative/reconstructive procedures compared with open flap debridement (OFD), in terms of clinical and/or radiographic outcome parameters and tooth retention?”	1041
(Tsai et al. 2020)	Taiwan – Taiwan	*Journal of Clinical Periodontology*	PRISMA-NMA	16	Ministry of Science & Technology in Taiwan	60	To update a previous network meta-analysis comparing the efficacyof periodontal regenerative therapies on the treatment of infrabony lesions	NR
(Cairo et al. 2020)	Italy – Italy	*Journal of Clinical Periodontology*	PRISMA	40	University of Michigan Periodontal Graduate Student Research Fund	26	To evaluate effect of different flap designs and graft materials for root coverage, in terms of aesthetics, patient satisfaction and self-reported morbidity	867
(Wang et al. 2020)	Taiwan – Taiwan	*Journal of Clinical Periodontology*	PRISMA	11	National Taiwan University Hospital; Ministry of Science & Technology, Taiwan	22	to evaluate the ef-ficacy of adjunctive locally delivered antimicrobials, compared to subgingival instru-mentation alone or plus a placebo	523
(Barbato et al. 2016)	Italy – Italy	*Journal of Periodontology*	PRISMA	30	NR	16	To evaluate and synthesize scientific evidence on the effect of surgical interventions for removal of mandibular third molar (M3M) on periodontal healing of adjacent mandibular second molar	509
(Kotsakis et al. 2018)	USA – USA	*Journal of Clinical Periodontology*	PRISMA	46	NIDCR grant; Waterpik Inc.	22	The aim of this study was to assess the comparative efficacy of IOH aids using Bayesian Network MetaAnalysis	NR
(Barootchi et al. 2020)	USA – USA	*Journal of Periodontology*	PRISMA-NMA	48	University of Michigan Periodontal Graduate Student Research Fund	105	To investigate the effect of PMT as it relates to root coverage and non-root coverage techniques on the periodontal conditions of natural teeth	NR
(Tavelli et al. 2021)	USA – USA	*Journal of Periodontology*	PRISMA-NMA	87	University of Michigan Periodontal Graduate Student Research Fund	23	To assess the efficacy of PSPM therapy in augmenting PSP (in terms of KMW, MT, and STH) and in promoting peri-implant health	1573+
(Sgolastra et al. 2021)	Italy – Italy	*Journal of Periodontal Research*	PRISMA-NMA	6	NR	21	To assess the efficacy of systemic anti-infective agents as an adjunct to SRP in the treatment of chronic periodontitis	1344
(Barbato et al. 2020)	Italy – Italy	*Clinical Oral Investigations*	PRISMA-NMA	15	NR	9	To explore the efficacy of different minimal invasive surgical (MIS) and nonsurgical (MINST) approaches for the treatment of intra-bony defect in terms of clinical attachment level (CAL) gain and periodontal pocket depth (PPD) reduction	244
(Moraschini et al. 2020)	Brazil – Brazil	*Clinical Oral Investigations*	PRISMA-NMA	8	No funding	27	To conduct a network comparison of the clinical effect of connective tissue graft (CTG) substitutes on the treatment of gingival recessions using coronally advanced flap	1286
(Pesce et al. 2022)	Italy – Italy	*Clinical Oral Investigations*	PRISMA-NMA	4	Università degli Studi di Genova	5	To compare periodontal indices in three categories of patients: traditional cigarette smokers (TS), e-cigarette smokers (ES), and nonsmokers (NS)	512
(Chambrone et al. 2022)	Brazil – Brazil	*Journal of Periodontology*	PRISMA	9	NR	45	To assess the efficacy of biologics in the context of PPS	396
(Chambrone et al. 2022)	Brazil – Brazil	*Journal of Periodontology*	PRISMA	3	BioHorizons (Birmingham, Alabama); Geistlich Biomaterials (Princeton, New Jersey); Lynch Biologics (Franklin, Tennessee); & Straumann USA (Andover, Massachusetts).	38	To assess the efficacy of a bilaminar root coverage technique con-sisting of the combination of an autogenous subepithelial connective tissue graft(SCTG) and a coronally advanced flap (CAF) compared with the five most indi-cated alternative approaches for the treatment of single gingival recession defects(GRD)	830
(Martins et al. 2022)	Brazil – Brazil	*Journal of Clinical Periodontology*	PRISMA	2	No funding received	22	To evaluate the efficacy of different techniques to seal the alveolus (flap advance-ment [FA], open healing with barrier [OHB], and open healing without barrier [OHNB]) during alveolar ridge preservation (ARP)	1262
(Mendonca et al. 2024)	Lisbon - Lisbon	*BMC Oral Health*	PRISMA-NMA	0	No funding received	33	In adult patients with periodontitis and good general health, what is the effect of the combination of PMPR and different existing probiotics in comparison with PMPR alone on probing pocket depth (PPD) reduction and clinical attachment level (CAL) gain?	NR
(Papageorgiou et al. 2016)	Germany - Germany	*Journal of Dentistry*	PRISMA-NMA	74	No funding received	45	This systematic review compared the histomorphometry effectiveness of bone grafts in an evidence based manner	852
(Hu et al. 2021)	Chine - China	*Lasers in medical Science*	PRISMA	10	National Natural Science Foundation of China	11	The present network meta-analysis addresses this gap and should provide guidance for dentists with respect to choosing the most suitable laser treatments for peri-implantitis/	NR
(Al-Moraissi et al. 2019)	Yemen - Yemen	*Journal of Oral and Maxillofacial Surgery*	PRISMA-NMA	31	NR	20	to identify the best rehabilitation method for patients with a posterior atrophic maxilla with 4 to 8 mm of alveolar ridge in relation to implant and prosthesis failure rates, marginal bone changes, and intra- and postoperative complications	770

Abbreviations = NR: not reported; NMA: network meta-analysis; PRISMA: Preferred Reporting Items for Systematic Reviews and Meta-Analyses; PRISMA-NMA: Preferred Reporting Items for Systematic Reviews and Meta-Analyses for Network Meta-Analyses; PRISMA-IPD: Preferred Reporting Items for Systematic Reviews and Meta-Analyses for Individual Participant Data.

All of the NMAs that were included for the study were published between 2010 and 2022, with a disproportionate number of papers published in 2022 ( *n* = 9, or 25%). The majority of the corresponding and the first authors of the included NMAs were affiliated with institutions belonging to Italy ( *n* = 11; 31%). Most of the NMAs were published in the *Journal of Clinical Periodontology* ( *n* = 19; 53%). The reporting guidelines employed for the NMA protocol varied: 17 articles (47%) used the PRISMA-NMA statement; 14 articles (39%) used the PRISMA statement; and 1 study (3%) used the PRISMA-Individual Participant Data statement. The top-cited article, as per the Scopus, was “A Bayesian network meta-analysis on comparisons of enamel matrix derivatives, guided tissue regeneration and their combined therapies” with 104 citations. 28 Twenty-four NMAs (67%) reported the funding source, five articles (14%) mentioned that no funding was received, and seven studies (19%) did not mention any details on the funding source. The number of primary studies incorporated in the NMAs exhibited substantial variation, spanning from 5 studies to 105 studies.

### Reporting Quality Outcomes

Table 2 depicts the overall reporting quality of the individual items present in the PRISMA-NMA statement. After evaluating the NMAs' compliance utilizing the 27-item PRISMA-NMA statement, 5 of the 39 studies complied with all 27 items. In summary, all 40 NMAs ranged between 87.5 and 100% compliance (i.e., high quality of reporting [≥ 75th percentile]). Supplementary Table S2 (available in the online version only) shows the complete tabular data of the individual NMAs and their compliance/noncompliance with the PRISMA-NMA checklist.

**Table 2:** Reporting quality evaluation of the included studies

Section and topic	Item no.	Checklist item	Reported (%)	Not reported (%)
Title	1	Title	100	0
Abstract	2	Structured summary	100	0
Introduction	3	Rationale	100	0
	4	Objectives	78	22
Methodology	5	Protocol and registration	53	47
	6	Eligibility criteria	100	0
	7	Information sources	100	0
	8	Search	100	0
	9	Study selection	100	0
	10	Data collection protocol	100	0
	11	Data items	100	0
	S1	Geometry of the network	100	0
	12	Risk of bias within individual studies	100	0
	13	Summary measures	100	0
	14	Planned methods of analysis	100	0
	S2	Assessment of inconsistency	100	0
	15	Risk of bias across studies	100	0
	16	Additional analyses	100	0
Results	17	Study selection	100	0
	S3	Presentation of network structure	94	6
	S4	Summary of network geometry	53	47
	18	Study features	100	0
	19	Risk of bias within studies	100	0
	20	Results of individual studies	100	0
	21	Synthesis of results	100	0
	S5	Exploration for inconsistency	100	0
	22	Risk of bias across studies	100	0
	23	Results of additional analyses	100	0
Discussion	24	Summary of evidence	100	0
	25	Limitations	97	3
	26	Conclusions	100	0
Funding	27	Funding	81	19

**Fig. 2:**
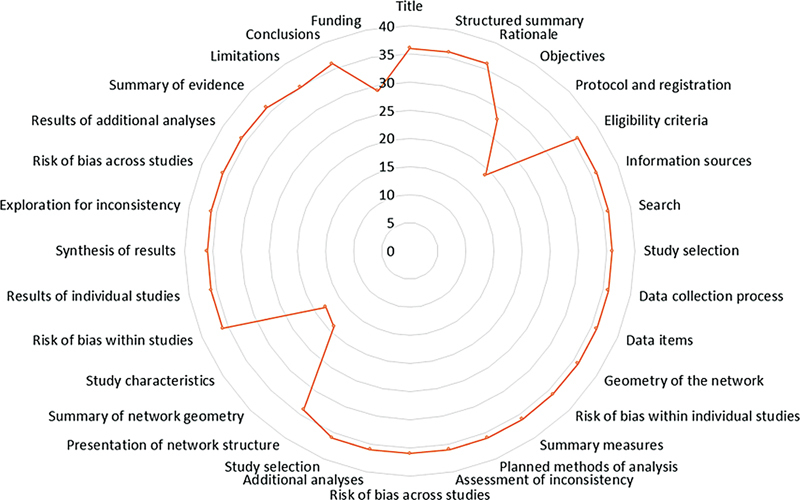
Radar map depicting the Preferred Reporting Items for Systematic Reviews and Meta-Analyses for Network Meta-Analyses (PRISMA-NMA) items reporting quality of the included studies.

Fig. 2 depicts the number of included NMAs sufficiently reported for the individual items. The following items were reported by all of the 39 included NMAs: (1) title; (2) structured summary; (3) rationale; (4) eligibility criteria; (5) information sources; (6) search; (7) study selection ( *methods* ); (8) data collection process; (9) data items; (10) the geometry of the network; (11) risk of bias (RoB) within individual studies ( *methods* ); (12) summary measures; (13) planned methods of analysis; (14) assessment of inconsistency; (15) RoB across studies; (16) additional analyses; (17) study selection ( *result* s); (18) study characteristics; (19) RoB within studies ( *results* ); (20) results of individual studies ( *results* ); (21) synthesis of results; (22) exploration of inconsistency; (23) RoB across studies ( *results* ); (24) results of additional analyses; (25) a summary of the evidence; and (26) conclusions. The limitations, presentation of network structure (results), funding, and objectives (methods) were reported in 97, 94, 81, and 78% of the NMAs, respectively. Notably absent from 53% of the NMAs were the components concerning the protocol and registration, as well as the overview of network geometry.

## Discussion

This analysis aimed to assess the quality of reporting of NMAs published in periodontology and implantology to assess if the studies adhered sufficiently to the PRISMA reporting items outlined in the SR protocol based on the PRISMA statement. 20 The findings of this analysis revealed an overall high reporting quality of around 96% established on the PRISMA-NMA checklist. Till today, this is the only study that found a high reporting quality of NMAs published in any field according to the PRISMA-NMA checklist. Recently, several such studies have been performed; however, all of them found low to moderate reporting quality of NMAs published in domains including dentistry, 14 16 acupuncture, 29 30 31 Chinese medicine, 32 physical therapy, 33 and knowledge synthesis. 34

In the present analysis, the SR of the literature identified 49 eligible articles until February 2023. The earliest publication year according to Scopus and Web of Science was 2010, and 51% ( *n* = 21/39) NMAs were published over the last 3 years, that is, 2020, 2021, and 2022. This indicates the establishment of NMA as a knowledge synthesis discipline. Similarly, around 83% of NMAs were published after 2015, pointing to a swift rise in the number of NMAs corresponding with the implementation of the PRISMA-NMA guidelines (i.e., 2015). Moreover, the Bayesian method was used in around 44% of the NMAs, and this might be because this method not only permits flexible modeling and efficient data integration but also permits the resulting posterior probability to rank all treatments included in the comparison according to their merit. 35

Around one-third of the included NMAs did not report the objective of the review according to the PICO (Population, Intervention, Comparison, Outcome) format. 36 The previously conducted SRs reported comparable reporting of this item (objectives) in the NMAs published related to acupuncture and moxibustion (76%), 31 better reporting outcomes in the NMAs published related to endodontology (100%) 14 37 and acupuncture (100%), 29 and poorer reporting in NMAs published related to general dentistry (68%), 16 physical therapy (68%), 33 and acupuncture (57%). 30 PICO is extensively adopted to formulate clinical questions for retrieving publications from the literature. 38 The PICO format is specialized to facilitate breaking down the requirement for evidence into searchable search terms and to develop answerable research questions. 39 A study by Schardt et al 40 found that the application of the PICO format can enhance publications searched against MEDLINE (PubMed). Nevertheless, owing to high requirements for dental/medical field knowledge and technical skills, clinicians, researchers, and the general public who need to search for evidence might find it either challenging to learn or time-consuming to add to their busy clinical workflow. 36 However, the recent PRISMA (2020) statement recommends the formulation of the research question based on the PICO framework. 19 Moreover, it will be beneficial for the readers if the title of NMA is representative of the PICO format since it gives the required information regarding the scope of the study. 41

The least reported item among the included NMAs was the summary of network geometry (53%), which is comparable to the findings of the study conducted previously regarding general dentistry (53%), 16 however, better than the outcomes of the study regarding endodontology (17%). 14 Moreover, a great deal of variation has been found regarding the reporting of this item in NMAs published on the topic of acupuncture (i.e., 3, 38, and 70%) 29 30 31 and Chinese medicine (50%). 32 Given that the summary of network geometry provides insights into the adequacy or inadequacy of intervention comparisons, it is crucial for readers to understand how both direct and indirect evidence contributed to the foundation of the NMA. 20

Another item that was found to be low to moderately reported in the present analysis was the protocol and registration of NMAs (53%). The misconception that protocol registration lacks benefits or is a time-consuming process has been disproven. 42 One primary drawback linked to noncompliance with protocol registration is the increased likelihood of duplicating the research question, a key aspect that protocol registration aims to address as one of its initial objectives. 43 Furthermore, several positives might be associated with adherence to protocol registration including (1) offering the reader an essential tool for preventing outcome reporting bias and, resultantly, publication bias and (2) enhancing indirectly the overall methodological and reporting quality of the NMA. 44 Before performing an NMA, investigators should conduct a scanning of the domain for any completed or ongoing NMAs on the same topic. 45 Nevertheless, several investigators do not publicly publish or even register the protocols of their ongoing NMAs, hence, contributing to the issue of duplication. 46 As a result, the imperative for NMA protocol registration has emerged, necessitating attention from decision-makers and guideline formulators. 42 Dos Santos et al 44 reported a positive influence on the final report quality of SRs in dentistry when the protocol was registered beforehand. Similarly, a study conducted by Sideri et al 47 48 evaluated SRs pertaining to orthodontics and found that a small proportion of SRs were registered in the International Prospective Register of Systematic Reviews (PROSPERO), nevertheless, the registered SRs had higher methodological quality as compared with the nonregistered ones.

It is imperative to recognize the strengths of the present analysis. First, to our knowledge, this analysis represents the initial attempt to evaluate the adequacy of reporting quality in NMAs associated with periodontology and implantology, as outlined by the PRISMA-NMA statement. Furthermore, for minimizing bias, two investigators performed the appraisal of the individual studies. Additionally, this is the most up-to-date and the largest analysis of published NMAs compiled to date in dentistry. The number of NMAs included in the present analysis was greater than the previously conducted studies in general dentistry, 44 endodontology, 14 37 acupuncture and moxibustion, 31 and physical therapy, 33 which included 21, 12, 29, and 19 NMAs, respectively. Additionally, to give a thorough review of periodontology and implantology, the current review consisted of NMAs with randomized and nonrandomized clinical trials and a variety of journals, including both periodontology and implantology journals and journals that do not focus on periodontology and implantology.

This study has certain limitations. While the authors applied appropriate methodology, they may have missed or excluded key details in the publication process. As reviewers, they could only assess what was explicitly reported. Additionally, the assessment focused exclusively on the reporting quality of the included NMAs, without evaluating their credibility or relevance. Ensuring the statistical validity of NMAs requires a thorough quality evaluation. Since the authors aimed to assess the reporting quality of NMAs in journals categorized under “Dentistry, Oral Surgery, and Medicine,” NMAs from nondental journals were excluded. Lastly, as the PRISMA-NMA guidelines are periodically updated, the findings of this study may evolve with new publications in periodontology and implantology.

It has been reported by Tonin et al 49 that the low quality of NMAs is because of poor methodology, and not reporting. Therefore, certain guidelines are needed solely for evaluating the methodological quality of NMAs that consider the particular assumptions of NMA. Consequently, such guidelines should encompass a description of the statistical analyses, network geometry presentation, summary of network structure, inconsistency in evaluation, any supplementary analyses conducted, and ranking order presentation. 49 It is recommended that the PRISMA-NMA statement should be utilized widely by authors and be adopted by a broader range of dentistry journals, especially the core journals of periodontology and implantology. The authors, reviewers, and editors are encouraged to use the PRISMA-NMA checklist regularly for writing, evaluating, and publishing their outcomes from NMAs, emphasizing the items that are not adequately reported including the protocol and registration and the summary of network geometry.

In brief, NMAs are challenging and complex, however, if well performed, they can yield the highest evidence level in comparative effectiveness research. To achieve this, collaborative efforts between statisticians and methodologists experienced in NMAs, experts in the conduct of SRs, and clinicians are required. Moreover, international efforts are required for encouraging reviewers and authors to adhere to the existing guidelines for limiting the publication of low-quality NMAs.

## Conclusion

The results of this critical analysis showed that the NMAs published in the fields of implantology and periodontology had good reporting quality. Nevertheless, several shortcomings were identified concerning the PRISMA-NMA items' reporting quality. These shortcomings include protocol registration, formulating the research question using the PICO format, and describing the network geometry.
