# Supplementary material for: Critical Analysis of Reporting Quality of Network Meta-Analyses in Periodontology and Implantology
Source: Eur J Dent. 2025 Mar 12;19(3):551–62. doi: 10.1055/s-0044-1801304 (PMC12182410; doi:10.1055/s-0044-1801304)
Supplement: Supplementary file 1 — Supplementary Material [file 10-1055-s-0044-1801304-s2453585.pdf]

Supplementary Table S1 Checklist of items to include when reporting a systematic review involving a network meta-analysis

Section/Topic	Item no.[a]	Checklist item[b]	Reported on page no.
**TITLE**			
Title	1	Identify the report as a systematic review *incorporating a network meta-analysis (or related form of meta-analysis).*	
**ABSTRACT**			
Structured summary	2	Provide a structured summary including, as applicable:<br>Background: main objectives.<br>Methods: data sources; study eligibility criteria, participants, and interventions; study appraisal; and *synthesis methods, such as network meta-analysis.*<br>Results: number of studies and participants identified; summary estimates with corresponding confidence/credible intervals; *treatment rankings may also be discussed. Authors may choose to summarize pairwise comparisons against a chosen treatment included in their analyses for brevity.*<br>Discussion/Conclusions: limitations; conclusions and implications of findings.<br>Other: primary source of funding; systematic review registration number with registry name.	
**INTRODUCTION**			
Rationale	3	Describe the rationale for the review in the context of what is already known, *including mention of why a network meta-analysis has been conducted.*	
Objectives	4	Provide an explicit statement of questions being addressed, with reference to participants, interventions, comparisons, outcomes, and study design (PICOS).	
**METHODS**			
Protocol and registration	5	Indicate whether a review protocol exists and if and where it can be accessed (e.g., Web address); and, if available, provide registration information, including registration number.	
Eligibility criteria	6	Specify study characteristics (e.g., PICOS, length of follow-up) and report characteristics (e.g., years considered, language, publication status) used as criteria for eligibility, giving rationale.<br>*Clearly describe eligible treatments included in the treatment network, and note whether any have been clustered or merged into the same node (with justification).*	
Information sources	7	Describe all information sources (e.g., databases with dates of coverage, contact with study authors to identify additional studies) in the search and date last searched.	
Search	8	Present full electronic search strategy for at least one database, including any limits used, such that it could be repeated.	
Study selection	9	State the process for selecting studies (i.e., screening, eligibility, included in systematic review, and, if applicable, included in the meta-analysis).	
Data collection process	10	Describe method of data extraction from reports (e.g., piloted forms, independently, in duplicate) and any processes for obtaining and confirming data from investigators.	
Data items	11	List and define all variables for which data were sought (e.g., PICOS, funding sources) and any assumptions and simplifications made.	

(*Continued*)

 © 2025. The Author(s).

Supplementary Table S1 (*Continued*)

Section/Topic	Item no.[a]	Checklist item[b]	Reported on page no.
Geometry of the network	**S1**	Describe methods used to explore the geometry of the treatment network under study and potential biases related to it. This should include how the evidence base has been graphically summarized for presentation, and what characteristics were com piled and used to describe the evidence base to readers.	
Risk of bias within individual studies	12	Describe methods used for assessing risk of bias of individual studies (including specification of whether this was done at the study or outcome level), and how this information is to be used in any data synthesis.	
Summary measures	13	State the principal summary measures (e.g., risk ratio, difference in means). *Also describe the use of additional summary measures assessed, such as treatment rankings and surface under the cumulative ranking curve (SUCRA) values, as well as modified approaches used to present summary findings from meta-analyses.*	
Planned methods of analysis	14	Describe the methods of handling data and combining results of studies for each network meta-analysis. This should include, but not be limited to:<br>*Handling of multigroup trials;*<br>*Selection of variance structure;*<br>*Selection of prior distributions in Bayesian analyses; and*<br>*Assessment of model fit.*	
Assessment of inconsistency	**S2**	Describe the statistical methods used to evaluate the agreement of direct and indirect evidence in the treatment network(s) studied. Describe efforts taken to address its presence when found.	
Risk of bias across studies	15	Specify any assessment of risk of bias that may affect the cumulative evidence (e.g., publication bias, selective reporting within studies).	
Additional analyses	16	Describe methods of additional analyses if done, indicating which w ere prespecified. This may include, but not be limited to, the following:<br>Sensitivity or subgroup analyses;<br>Meta-regression analyses;<br>*Alternative formulations of the treatment network; and*<br>*Use of alternative prior distributions for Bayesian analyses (if applicable).*	
**RESULTS[c]**			
Study selection	17	Give numbers of studies screened, assessed for eligibility, and included in the review, with reasons for exclusions at each stage, ideally with a flow diagram.	
Presentation of network geometry	**S3**	Provide a network graph of the included studies to enable visualization of the geometry of the treatment network.	
Summary of network geometry	**S4**	Provide a brief overview of characteristics of the treatment network. This may include commentary on the abundance of trials and randomized patients for the different interventions and pairwise comparisons in the network, gaps of evidence in the treatment network, and potential biases reflected by the network structure.	
Study characteristics	18	For each study, present characteristics for which data were extracted (e.g., study size, PICOS, follow-up period) and provide the citations.	
Risk of bias within studies	19	Present data on risk of bias of each study and, if available, any outcome level assessment.	

 © 2025. The Author(s).

Supplementary Table S1 (*Continued*)

Section/Topic	Item no.[a]	Checklist item[b]	Reported on page no.
Results of individual studies	20	For all outcomes considered (benefits or harms), present, for each study: 1) simple summary data for each intervention group, and 2) effect estimates and confidence intervals. *Modified approaches may be needed to deal with information from larger networks.*	
Synthesis of results	21	Present results of each meta-analysis done, including confidence/credible intervals. *In larger networks, authors may focus on comparisons versus a particular comparator (e.g., placebo or standard care), with full findings presented in an appendix. League tables and forest plots may be considered to summarize pairwise comparisons.* If additional summary measures were explored (such as treatment rankings), these should also be presented.	
Exploration for inconsistency	**S5**	Describe results from investigations of inconsistency. This may include such information as measures of model fit to compare consistency and inconsistency models, *P*-values from statistical tests, or summary of inconsistency estimates from different parts of the treatment network.	
Risk of bias across studies	22	Present results of any assessment of risk of bias across studies for the evidence base being studied	
Results of additional analyses	23	Give results of additional analyses, if done (e.g., sensitivity or subgroup analyses, meta-regression analyses, *alternative network geometries studied, alternative choice of prior distributions for Bayesian analyses, and so forth).*	
**DISCUSSION**			
Summary of evidence	24	Summarize the main findings, including the strength of evidence for each main outcome; consider their relevance to key groups (e.g., health care providers, researchers, and policymakers).	
Limitations	25	Discuss limitations at study and outcome level (e.g., risk of bias), and at review level (e.g., incomplete retrieval of identified research, reporting bias). *Comment on the validity of the assumptions, such as transitivity and consistency. Comment on any concerns regarding network geometry (e.g., avoidance of certain comparisons)*.	
Conclusions	26	Provide a general interpretation of the results in the context of other evidence, and implications for future research.	
**FUNDING**			
Funding	27	Describe sources of funding for the systematic review and other support (e.g., supply of data); role of funders for the systematic review. This should also include information regarding whether funding has been received from manufacturers of treatments in the network and/or whether some of the authors are content experts with professional conflicts of interest that could affect use of treatments in the network.	

Abbreviation: PRISMA, Preferred Reporting Items for Systematic Reviews and Meta-Analyses.
[a]Boldface indicates new items to this checklist.
[b]Text in italics indicates wording specific to reporting of network meta-analyses that has been added to guidance from the PRISMA statement.
[c]Authors may wish to plan for use of appendices to present all relevant information in full detail for items in this section.

 © 2025. The Author(s).

Supplementary Table S2 The reporting quality of the included studies according to the PRISMA-NMA checklist

Study (according to the list of included studies)																		
**Item**	**1**	**2**	**3**	**4**	**5**	**6**	**7**	**8**	**9**	**10**	**11**	**12**	**13**	**14**	**15**	**16**	**17**	**18**
1	Yes	Yes	Yes	Yes	Yes	Yes	Yes	Yes	Yes	Yes	Yes	Yes	Yes	Yes	Yes	Yes	Yes	Yes
2	Yes	Yes	Yes	Yes	Yes	Yes	Yes	Yes	Yes	Yes	Yes	Yes	Yes	Yes	Yes	Yes	Yes	Yes
3	Yes	Yes	Yes	Yes	Yes	Yes	Yes	Yes	Yes	Yes	Yes	Yes	Yes	Yes	Yes	Yes	Yes	Yes
4	Yes	Yes	Yes	Yes	Yes	No	Yes	Yes	No	Yes	No	No	No	Yes	Yes	Yes	No	Yes
5	No	Yes	Yes	No	No	No	Yes	Yes	No	No	No	No	Yes	No	No	Yes	No	No
6	Yes	Yes	Yes	Yes	Yes	Yes	Yes	Yes	Yes	Yes	Yes	Yes	Yes	Yes	Yes	Yes	Yes	Yes
7	Yes	Yes	Yes	Yes	Yes	Yes	Yes	Yes	Yes	Yes	Yes	Yes	Yes	Yes	Yes	Yes	Yes	Yes
8	Yes	Yes	Yes	Yes	Yes	Yes	Yes	Yes	Yes	Yes	Yes	Yes	Yes	Yes	Yes	Yes	Yes	Yes
9	Yes	Yes	Yes	Yes	Yes	Yes	Yes	Yes	Yes	Yes	Yes	Yes	Yes	Yes	Yes	Yes	Yes	Yes
10	Yes	Yes	Yes	Yes	Yes	Yes	Yes	Yes	Yes	Yes	Yes	Yes	Yes	Yes	Yes	Yes	Yes	Yes
11	Yes	Yes	Yes	Yes	Yes	Yes	Yes	Yes	Yes	Yes	Yes	Yes	Yes	Yes	Yes	Yes	Yes	Yes
S1	Yes	Yes	Yes	Yes	Yes	Yes	Yes	Yes	Yes	Yes	Yes	Yes	Yes	Yes	Yes	Yes	Yes	Yes
12	Yes	Yes	Yes	Yes	Yes	Yes	Yes	Yes	Yes	Yes	Yes	Yes	Yes	Yes	Yes	Yes	Yes	Yes
13	Yes	Yes	Yes	Yes	Yes	Yes	Yes	Yes	Yes	Yes	Yes	Yes	Yes	Yes	Yes	Yes	Yes	Yes
14	Yes	Yes	Yes	Yes	Yes	Yes	Yes	Yes	Yes	Yes	Yes	Yes	Yes	Yes	Yes	Yes	Yes	Yes
S2	Yes	Yes	Yes	Yes	Yes	Yes	Yes	Yes	Yes	Yes	Yes	Yes	Yes	Yes	Yes	Yes	Yes	Yes
15	Yes	Yes	Yes	Yes	Yes	Yes	Yes	Yes	Yes	Yes	Yes	Yes	Yes	Yes	Yes	Yes	Yes	Yes
16	Yes	Yes	Yes	Yes	Yes	Yes	Yes	Yes	Yes	Yes	Yes	Yes	Yes	Yes	Yes	Yes	Yes	Yes
17	Yes	Yes	Yes	Yes	Yes	Yes	Yes	Yes	Yes	Yes	Yes	Yes	Yes	Yes	Yes	Yes	Yes	Yes
S3	Yes	Yes	Yes	Yes	Yes	No	Yes	Yes	Yes	Yes	Yes	Yes	No	Yes	Yes	Yes	Yes	Yes
S4	No	No	No	No	No	No	No	No	Yes	Yes	Yes	No	No	No	Yes	Yes	Yes	Yes
18	Yes	Yes	Yes	Yes	Yes	Yes	Yes	Yes	Yes	Yes	Yes	Yes	Yes	Yes	Yes	Yes	Yes	Yes
19	Yes	Yes	Yes	Yes	Yes	Yes	Yes	Yes	Yes	Yes	Yes	Yes	Yes	Yes	Yes	Yes	Yes	Yes
20	Yes	Yes	Yes	Yes	Yes	Yes	Yes	Yes	Yes	Yes	Yes	Yes	Yes	Yes	Yes	Yes	Yes	Yes
21	Yes	Yes	Yes	Yes	Yes	Yes	Yes	Yes	Yes	Yes	Yes	Yes	Yes	Yes	Yes	Yes	Yes	Yes
S5	Yes	Yes	Yes	Yes	Yes	Yes	Yes	Yes	Yes	Yes	Yes	Yes	Yes	Yes	Yes	Yes	Yes	Yes
22	Yes	Yes	Yes	Yes	Yes	Yes	Yes	Yes	Yes	Yes	Yes	Yes	Yes	Yes	Yes	Yes	Yes	Yes
23	Yes	Yes	Yes	Yes	Yes	Yes	Yes	Yes	Yes	Yes	Yes	Yes	Yes	Yes	Yes	Yes	Yes	Yes
24	Yes	Yes	Yes	Yes	Yes	Yes	Yes	Yes	Yes	Yes	Yes	Yes	Yes	Yes	Yes	Yes	Yes	Yes
25	Yes	No	Yes	Yes	Yes	Yes	Yes	Yes	Yes	Yes	Yes	Yes	Yes	Yes	Yes	Yes	Yes	Yes
26	Yes	Yes	Yes	Yes	Yes	Yes	Yes	Yes	Yes	Yes	Yes	Yes	Yes	Yes	Yes	Yes	Yes	Yes
27	No	Yes	Yes	Yes	Yes	Yes	Yes	Yes	Yes	Yes	Yes	Yes	Yes	Yes	Yes	Yes	Yes	Yes
**Study (according to the list of included studies)**																		
**Item**	**19**	**20**	**21**	**22**	**23**	**24**	**25**	**26**	**27**	**28**	**29**	**30**	**31**	**32**	**33**	**34**	**35**	**36**
1	Yes	Yes	Yes	Yes	Yes	Yes	Yes	Yes	Yes	Yes	Yes	Yes	Yes	Yes	Yes	Yes	Yes	Yes
2	Yes	Yes	Yes	Yes	Yes	Yes	Yes	Yes	Yes	Yes	Yes	Yes	Yes	Yes	Yes	Yes	Yes	Yes
3	Yes	Yes	Yes	Yes	Yes	Yes	Yes	Yes	Yes	Yes	Yes	Yes	Yes	Yes	Yes	Yes	Yes	Yes
4	Yes	Yes	Yes	No	No	Yes	Yes	Yes	Yes	Yes	Yes	Yes	Yes	Yes	Yes	Yes	Yes	Yes
5	Yes	Yes	Yes	No	No	Yes	No	Yes	No	Yes	Yes	Yes	No	Yes	Yes	Yes	Yes	Yes
6	Yes	Yes	Yes	Yes	Yes	Yes	Yes	Yes	Yes	Yes	Yes	Yes	Yes	Yes	Yes	Yes	Yes	Yes
7	Yes	Yes	Yes	Yes	Yes	Yes	Yes	Yes	Yes	Yes	Yes	Yes	Yes	Yes	Yes	Yes	Yes	Yes
8	Yes	Yes	Yes	Yes	Yes	Yes	Yes	Yes	Yes	Yes	Yes	Yes	Yes	Yes	Yes	Yes	Yes	Yes

 © 2025. The Author(s).

Supplementary Table S2 (*Continued*)

Study (according to the list of included studies)																		
**Item**	**1**	**2**	**3**	**4**	**5**	**6**	**7**	**8**	**9**	**10**	**11**	**12**	**13**	**14**	**15**	**16**	**17**	**18**
**9**	Yes	Yes	Yes	Yes	Yes	Yes	Yes	Yes	Yes	Yes	Yes	Yes	Yes	Yes	Yes	Yes	Yes	Yes
**10**	Yes	Yes	Yes	Yes	Yes	Yes	Yes	Yes	Yes	Yes	Yes	Yes	Yes	Yes	Yes	Yes	Yes	Yes
**11**	Yes	Yes	Yes	Yes	Yes	Yes	Yes	Yes	Yes	Yes	Yes	Yes	Yes	Yes	Yes	Yes	Yes	Yes
**S1**	Yes	Yes	Yes	Yes	Yes	Yes	Yes	Yes	Yes	Yes	Yes	Yes	Yes	Yes	Yes	Yes	Yes	Yes
**12**	Yes	Yes	Yes	Yes	Yes	Yes	Yes	Yes	Yes	Yes	Yes	Yes	Yes	Yes	Yes	Yes	Yes	Yes
**13**	Yes	Yes	Yes	Yes	Yes	Yes	Yes	Yes	Yes	Yes	Yes	Yes	Yes	Yes	Yes	Yes	Yes	Yes
**14**	Yes	Yes	Yes	Yes	Yes	Yes	Yes	Yes	Yes	Yes	Yes	Yes	Yes	Yes	Yes	Yes	Yes	Yes
**S2**	Yes	Yes	Yes	Yes	Yes	Yes	Yes	Yes	Yes	Yes	Yes	Yes	Yes	Yes	Yes	Yes	Yes	Yes
**15**	Yes	Yes	Yes	Yes	Yes	Yes	Yes	Yes	Yes	Yes	Yes	Yes	Yes	Yes	Yes	Yes	Yes	Yes
**16**	Yes	Yes	Yes	Yes	Yes	Yes	Yes	Yes	Yes	Yes	Yes	Yes	Yes	Yes	Yes	Yes	Yes	Yes
**17**	Yes	Yes	Yes	Yes	Yes	Yes	Yes	Yes	Yes	Yes	Yes	Yes	Yes	Yes	Yes	Yes	Yes	Yes
**S3**	Yes	Yes	Yes	Yes	Yes	Yes	Yes	Yes	Yes	Yes	Yes	Yes	Yes	Yes	Yes	Yes	Yes	Yes
**S4**	Yes	Yes	Yes	Yes	No	No	Yes	No	Yes	No	No	Yes	Yes	No	Yes	Yes	Yes	Yes
**18**	Yes	Yes	Yes	Yes	Yes	Yes	Yes	Yes	Yes	Yes	Yes	Yes	Yes	Yes	Yes	Yes	Yes	Yes
**19**	Yes	Yes	Yes	Yes	Yes	Yes	Yes	Yes	Yes	Yes	Yes	Yes	Yes	Yes	Yes	Yes	Yes	Yes
**20**	Yes	Yes	Yes	Yes	Yes	Yes	Yes	Yes	Yes	Yes	Yes	Yes	Yes	Yes	Yes	Yes	Yes	Yes
**21**	Yes	Yes	Yes	Yes	Yes	Yes	Yes	Yes	Yes	Yes	Yes	Yes	Yes	Yes	Yes	Yes	Yes	Yes
**S5**	Yes	Yes	Yes	Yes	Yes	Yes	Yes	Yes	Yes	Yes	Yes	Yes	Yes	Yes	Yes	Yes	Yes	Yes
**22**	Yes	Yes	Yes	Yes	Yes	Yes	Yes	Yes	Yes	Yes	Yes	Yes	Yes	Yes	Yes	Yes	Yes	Yes
**23**	Yes	Yes	Yes	Yes	Yes	Yes	Yes	Yes	Yes	Yes	Yes	Yes	Yes	Yes	Yes	Yes	Yes	Yes
**24**	Yes	Yes	Yes	Yes	Yes	Yes	Yes	Yes	Yes	Yes	Yes	Yes	Yes	Yes	Yes	Yes	Yes	Yes
**25**	Yes	Yes	Yes	Yes	Yes	Yes	Yes	Yes	Yes	Yes	Yes	Yes	Yes	Yes	Yes	Yes	Yes	Yes
**26**	Yes	Yes	Yes	Yes	Yes	Yes	Yes	Yes	Yes	Yes	Yes	Yes	Yes	Yes	Yes	Yes	Yes	Yes
**27**	No	No	Yes	Yes	Yes	Yes	Yes	No	Yes	Yes	Yes	No	No	Yes	Yes	No	Yes	Yes

Study (according to the list of included studies)				
Item	37	38	39	40
1	Yes	Yes	Yes	Yes
2	Yes	Yes	Yes	Yes
3	Yes	Yes	Yes	Yes
4	Yes	No	Yes	Yes
5	Yes	No	Yes	Yes
6	Yes	Yes	Yes	Yes
7	Yes	Yes	Yes	Yes
8	Yes	Yes	Yes	Yes
9	Yes	Yes	Yes	Yes
10	Yes	Yes	Yes	Yes
11	Yes	Yes	Yes	Yes
12	Yes	Yes	Yes	Yes
13	Yes	Yes	Yes	Yes
14	Yes	Yes	Yes	Yes
S2	Yes	Yes	Yes	Yes

(*Continued*)

 © 2025. The Author(s).

(*Continued*)

15	Yes	Yes	Yes	Yes
16	Yes	Yes	Yes	Yes
17	Yes	Yes	Yes	Yes
S3	Yes	Yes	Yes	Yes
S4	Yes	Yes	Yes	Yes
18	Yes	No	Yes	Yes
19	Yes	Yes	No	Yes
20	Yes	Yes	Yes	Yes
21	Yes	Yes	Yes	Yes
S5	Yes	Yes	Yes	Yes
22	Yes	Yes	Yes	Yes
23	Yes	Yes	Yes	Yes
24	Yes	Yes	Yes	Yes
25	Yes	Yes	Yes	Yes
26	Yes	Yes	Yes	Yes
27	Yes	Yes	Yes	Yes

Abbreviation: PRISMA-NMA, Preferred Reporting Items for Systematic Reviews and Meta-Analyses for Network Meta-Analyses.

Search Strings for PubMed, Scopus, EMBASE, and Web of Science

Database	Search String
PubMed	(“network meta-analysis”[Title] OR “indirect meta-analysis”[Title] OR “mixed treatment meta-analysis”[Title] OR “mixed intervention meta-analysis”[Title] OR “multiple treatment comparison meta-analysis”[Title] OR “multiple intervention comparison meta-analysis”[Title] OR “bayes meta-analysis”[Title] OR “indirect comparison”[Title]) AND (“periodontics” OR “periodontal” OR “periodontitis” OR “dental implants”)
**Database**	**Search String**
Scopus	TITLE(“network meta-analysis” OR “indirect meta-analysis” OR “mixed treatment meta-analysis” OR “mixed intervention meta-analysis” OR “multiple treatment comparison meta-analysis” OR “multiple intervention comparison meta-analysis” OR “bayes meta-analysis” OR “indirect comparison”) AND TITLE(“periodontics” OR “periodontal” OR “periodontitis” OR “dental implants”)
**Database**	**Search String**
EMBASE	(’network meta-analysis’:ti OR ’indirect meta-analysis’:ti OR ’mixed treatment meta-analysis’:ti OR ’mixed intervention meta-analysis’:ti OR ’multiple treatment comparison meta-analysis’:ti OR ’multiple intervention comparison meta-analysis’:ti OR ’bayes meta-analysis’:ti OR ’indirect comparison’:ti) AND (’periodontics’:ti ’periodontal’:ti periodontitis’:ti dental implants’:ti)
**Database**	**Search String**
Web of Science	TI = (“network meta-analysis” OR “indirect meta-analysis” OR “mixed treatment meta-analysis” OR “mixed intervention meta-analysis” OR “multiple treatment comparison meta-analysis” OR “multiple intervention comparison meta-analysis” OR “bayes meta-analysis” OR “indirect comparison”) AND TI = (“periodontics” OR “periodontal” OR “periodontitis” OR “dental implants”)

 © 2025. The Author(s).

List Of Included Studies

1 Aldhohrah T, Mashrah MA, Wang Y. Effect of 2-implant mandibular overdenture with different attachments and loading protocols on peri-implant health and prosthetic complications: a systematic review and network meta-analysis. J Prosthet Dent 2022;127 (06):832–844

2 Camps-Font O, Rubianes-Porta L, Valmaseda-Castellón E, Jung RE, Gay-Escoda C, Figueiredo R. Comparison of external, internal flat-to-flat, and conical implant abutment connections for implant-supported prostheses: a systematic review and network meta-analysis of randomized clinical trials. J Prosthet Dent 2021

3 Hu M, Chen J, Pei X, Han J, Wang J. Network meta-analysis of survival rate and complications in implant-supported single crowns with different abutment materials. J Dent 2019; 88:103115

4 Bi Y, Aldhohrah T, Mashrah MA, et al. Effects of attachment type and number of dental implants supporting mandibular overdenture on peri-implant health: a systematic review and network meta-analysis. J Prosthodont Res 2022;66(03):357–373

5 Severi M, Simonelli A, Farina R, et al. Effect of lateral bone augmentation procedures in correcting peri-implant bone dehiscence and fenestration defects: a systematic review and network meta-analysis. Clin Implant Dent Relat Res 2022;24(02):251–264

6 Faggion CM Jr, Chambrone L, Listl S, Tu YK. Network meta-analysis for evaluating interventions in implant dentistry: the case of peri-implantitis treatment. Clin Implant Dent Relat Res 2013;15(04): 576–588

7 Panda S, Khijmatgar S, Arbildo-Vega H, et al. Stability of biomaterials used in adjunct to coronally advanced flap: a systematic review and network meta-analysis. Clin Exp Dent Res 2022;8(01): 421–438

8 Li ZB, Li K, Ding T, Yang HQ, Du M, Yu Y. The effect of non-augmentative approaches in the surgical treatment of peri-implantitis: a systematic review and network meta-analysis. Int J Oral Maxillofac Implants 2022;37(02):270–282

9 Tu YK, Woolston A, Faggion CM Jr. Do bone grafts or barrier membranes provide additional treatment effects for infrabony lesions treated with enamel matrix derivatives? A network meta-analysis of randomized-controlled trials. J Clin Periodontol 2010; 37(01):59–79

10 Buti J, Baccini M, Nieri M, La Marca M, Pini-Prato GP. Bayesian network meta-analysis of root coverage procedures: ranking efficacy and identification of best treatment. J Clin Periodontol 2013;40(04):372–386

11 Tu YK, Needleman I, Chambrone L, Lu HK, Faggion CM Jr. A Bayesian network meta-analysis on comparisons of enamel matrix derivatives, guided tissue regeneration and their combination therapies. J Clin Periodontol 2012;39(03):303–314

12 Faggion CM Jr, Listl S, Frühauf N, Chang HJ, Tu YK. A systematic review and Bayesian network meta-analysis of randomized clinical trials on non-surgical treatments for peri-implantitis. J Clin Periodontol 2014;41(10):1015–1025

13 Rabelo CC, Feres M, Gonçalves C, et al. Systemic antibiotics in the treatment of aggressive periodontitis. A systematic review and a Bayesian network meta-analysis. J Clin Periodontol 2015;42(07): 647–657

14 Escribano M, Figuero E, Martín C, et al. Efficacy of adjunctive anti-plaque chemical agents: a systematic review and network meta-analyses of the Turesky modification of the Quigley and Hein plaque index. J Clin Periodontol 2016;43(12):1059–1073

15 Iocca O, Farcomeni A, Pardiñas Lopez S, Talib HS. Alveolar ridge preservation after tooth extraction: a Bayesian network meta-analysis of grafting materials efficacy on prevention of bone height and width reduction. J Clin Periodontol 2017;44(01): 104–114

16 John MT, Michalowicz BS, Kotsakis GA, Chu H. Network meta-analysis of studies included in the Clinical Practice Guideline on the nonsurgical treatment of chronic periodontitis. J Clin Periodontol 2017;44(06):603–611

17 Figuero E, Herrera D, Tobías A, et al. Efficacy of adjunctive anti-plaque chemical agents in managing gingivitis: a systematic review and network meta-analyses. J Clin Periodontol 2019;46 (07):723–739

18 Jepsen S, Gennai S, Hirschfeld J, Kalemaj Z, Buti J, Graziani F. Regenerative surgical treatment of furcation defects: a systematic review and Bayesian network meta-analysis of randomized clinical trials. J Clin Periodontol 2020;47(Suppl 22):352–374

19 Romandini M, De Tullio I, Congedi F, et al. Antibiotic prophylaxis at dental implant placement: Which is the best protocol? A systematic review and network meta-analysis. J Clin Periodontol 2019; 46(03):382–395

20 Slot DE, Valkenburg C, Van der Weijden GAF. Mechanical plaque removal of periodontal maintenance patients: a systematic review and network meta-analysis. J Clin Periodontol 2020;47 (Suppl 22):107–124

21 Stavropoulos A, Bertl K, Spineli LM, Sculean A, Cortellini P, Tonetti M. Medium- and long-term clinical benefits of periodontal regenerative/reconstructive procedures in intrabony defects: systematic review and network meta-analysis of randomized controlled clinical studies. J Clin Periodontol 2021;48(03): 410–430

22 Tsai SJ, Ding YW, Shih MC, Tu YK. Systematic review and sequential network meta-analysis on the efficacy of periodontal regenerative therapies. J Clin Periodontol 2020;47(09):1108–1120

23 Cairo F, Barootchi S, Tavelli L, et al. Aesthetic- and patient-related outcomes following root coverage procedures: a systematic review and network meta-analysis. J Clin Periodontol 2020;47(11): 1403–1415

24 Wang CY, Yang YH, Li H, et al. Adjunctive local treatments for patients with residual pockets during supportive periodontal care: a systematic review and network meta-analysis. J Clin Periodontol 2020;47(12):1496–1510

25 Barbato L, Kalemaj Z, Buti J, et al. Effect of surgical intervention for removal of mandibular third molar on periodontal healing of adjacent mandibular second molar: a systematic review and Bayesian network meta-analysis. J Periodontol 2016;87(03): 291–302

26 Kotsakis GA, Lian Q, Ioannou AL, Michalowicz BS, John MT, Chu H. A network meta-analysis of interproximal oral hygiene methods in the reduction of clinical indices of inflammation. J Periodontol 2018;89(05):558–570

27 Barootchi S, Tavelli L, Zucchelli G, Giannobile WV, Wang HL. Gingival phenotype modification therapies on natural teeth: a network meta-analysis. J Periodontol 2020;91(11):1386–1399

28 Tavelli L, Barootchi S, Avila-Ortiz G, Urban IA, Giannobile WV, Wang HL. Peri-implant soft tissue phenotype modification and its impact on peri-implant health: a systematic review and network meta-analysis. J Periodontol 2021;92(01):21–44

29 Sgolastra F, Petrucci A, Ciarrocchi I, Masci C, Spadaro A. Adjunctive systemic antimicrobials in the treatment of chronic periodontitis: a systematic review and network meta-analysis. J Periodontal Res 2021;56(02):236–248

30 Barbato L, Selvaggi F, Kalemaj Z, et al. Clinical efficacy of minimally invasive surgical (MIS) and non-surgical (MINST) treatments of periodontal intra-bony defect. A systematic review and network meta-analysis of RCT's. Clin Oral Investig 2020;24(03):1125–1135

31 Moraschini V, Calasans-Maia MD, Dias AT, et al. Effectiveness of connective tissue graft substitutes for the treatment of gingival recessions compared with coronally advanced flap: a network meta-analysis. Clin Oral Investig 2020;24(10):3395–3406

 © 2025. The Author(s).

32 Pesce P, Menini M, Ugo G, Bagnasco F, Dioguardi M, Troiano G. Evaluation of periodontal indices among non-smokers, tobacco, and e-cigarette smokers: a systematic review and network meta-analysis. Clin Oral Investig 2022;26(07):4701–4714
33 Chambrone L, Botelho J, Machado V, Mascarenhas P, Mendes JJ, Avila-Ortiz G. Does the subepithelial connective tissue graft in conjunction with a coronally advanced flap remain as the gold standard therapy for the treatment of single gingival recession defects? A systematic review and network meta-analysis. J Periodontol 2022;93(09):1336–1352
34 Chambrone L, Barootchi S, Avila-Ortiz G. Efficacy of biologics in root coverage and gingival augmentation therapy: an American Academy of Periodontology best evidence systematic review and network meta-analysis. J Periodontol 2022;93(12):1771–1802
35 Martins JR, Wagner TP, Vallim AC, et al. Comparison of the efficacy of different techniques to seal the alveolus during alveolar ridge preservation: meta-regression and network meta-analysis. J Clin Periodontol 2022;49(07):694–705
36 Mendonça CD, Mata ADSPD, Azevedo LFR, Marques JF, Silveira JML, Marques DNDS. Probiotics in the non-surgical treatment of periodontitis: a systematic review and network meta-analysis. BMC Oral Health 2024;24(01):1224
37 Papageorgiou SN, Papageorgiou PN, Deschner J, Götz W. Comparative effectiveness of natural and synthetic bone grafts in oral and maxillofacial surgery prior to insertion of dental implants: systematic review and network meta-analysis of parallel and cluster randomized controlled trials. J Dent 2016;48:1–8
38 Hu ML, Zheng G, Lin H, Li N, Zhao PF, Han JM. Network meta-analysis of the treatment efficacy of different lasers for peri-implantitis. Lasers Med Sci 2021;36(03):619–629
39 Al-Moraissi EA, Altairi NH, Abotaleb B, Al-Iryani G, Halboub E, Alakhali MS. What is the most effective rehabilitation method for posterior maxillas with 4 to 8 mm of residual alveolar bone height below the maxillary sinus with implant-supported prostheses? A frequentist network meta-analysis. J Oral Maxillofac Surg 2019; 77(01):70.e1–70.e33

List of Excluded Studies due to Nonavailability of Full Text

1 Li J, Gao W, Punja S, et al. Reporting quality of N-of-1 trials published between 1985 and 2013: a systematic review. J Clin Epidemiol 2016;76:57–64
2 Chambrone L, Barootchi S, Avila-Ortiz G. Efficacy of biologics in root coverage and gingival augmentation therapy: an American Academy of Periodontology best evidence systematic review and network meta-analysis. J Periodontol 2022;93(12):1771–1802
3 Martins JR, Wagner TP, Vallim AC, et al. Comparison of the efficacy of different techniques to seal the alveolus during alveolar ridge preservation: meta-regression and network meta-analysis. J Clin Periodontol 2022;49(07):694–705
4 Ye L, Mashrah MA, Ge L, et al. Network meta-analysis of platelet-rich fibrin in periodontal intrabony defects. J Oral Pathol Med 2023;52(03):206–215
5 Wu XY, Shi JY, Buti J, Lai HC, Tonetti MS. Buccal bone thickness and mid-facial soft tissue recession after various surgical approaches for immediate implant placement: a systematic review and network meta-analysis of controlled trials. J Clin Periodontol 2023;50(04):533–546
6 Tavelli L, Chen CJ, Barootchi S, Kim DM. Efficacy of biologics for the treatment of periodontal infrabony defects: an American Academy of Periodontology best evidence systematic review and network meta-analysis. J Periodontol 2022;93(12): 1803–1826

 © 2025. The Author(s).
